# Arbaeen in the Context of the COVID-19 Pandemic

**DOI:** 10.1017/dmp.2020.362

**Published:** 2020-10-02

**Authors:** Lara Hamdanieh, Abbas Ostadtaghizadeh

**Affiliations:** Department of Health in Emergencies and Disasters, School of Public Health, Tehran University of Medical Sciences, Tehran, Iran

**Keywords:** Arbaeen, mass gathering, COVID-19

The Arbaeen pilgrimage is an Islamic mass gathering (MG) that takes place in Iraq. It is estimated that 17 to 20 million people from different countries participate in this annual procession. Pilgrims walk for days from several Iraqi cities to reach Karbala on the day of Arbaeen. Different voluntary services, such as accommodation, food, and health, are provided to the pilgrims during the MG.^1^

Infectious diseases are the most common health threat with MGs.^1^ During Arbaeen, controlling infectious diseases is so challenging due to the high population density, non-resilient health infrastructure, limited control of infectious diseases, low participant perception about health risk factors, and ineffective health education. Also, in the absence of food safety supervision, gastrointestinal infections could occur.^2^ The Arbaeen MG can be a hub with the potential to disseminate the new coronavirus and exacerbate the scope of the coronavirus disease (COVID-19) pandemic. This MG and its associated impacts are a serious health threat, and ignoring this issue can lead to unprecedented health consequences. Whether or not the authorities decide to cancel Arbaeen, it is expected that many people from Iraq and other countries will participate in this religious ceremony during September to October 2020. Due to the spiritual purpose of the walk, some pilgrims might believe that there is low health risks in this MG and they won’t follow the protective measures.^1^

Therefore, national and international organizations should consider COVID-19 prevention and control measures during MGs a top priority on their agenda. This includes conducting a risk assessment,^3^ developing an emergency operations plan, and ensuring effective risk communication.^4^
